# Attending sporting mega events during COVID-19: mitigation and messaging at UK EURO 2020 matches

**DOI:** 10.1093/heapro/daac176

**Published:** 2023-01-07

**Authors:** Richard I Purves, Jordan Maclean, Claudio Rocha, Matthew Philpott, Niamh Fitzgerald, Joe Piggin, Kate Hunt

**Affiliations:** Institute for Social Marketing and Health, University of Stirling, Stirling, UK; Institute for Social Marketing and Health, University of Stirling, Stirling, UK; Faculty of Health Sciences and Sport, University of Stirling, Stirling, UK; European Healthy Stadia Network, Liverpool, UK; Institute for Social Marketing and Health, University of Stirling, Stirling, UK; Loughborough University, Loughborough, UK; Institute for Social Marketing and Health, University of Stirling, Stirling, UK

**Keywords:** Sport, mitigation, messaging, COVID-19, transmission

## Abstract

The UEFA EURO 2020 football tournament was one of the largest Sporting Mega Events (SMEs) to take place during the COVID-19 pandemic. Mitigating the risk of virus transmission requires a multi-layered approach for any large event, more so in this case due to staging the tournament across eleven host countries. Yet, little is known about COVID-19 risks and mitigation from attending an event of this scale and nature. We examined the implementation of mitigation and messaging at EURO 2020 matches hosted at venues in the UK. The tournament was postponed from the summer of 2020 and played in June and July of 2021. Structured observations were conducted by 11 trained fieldwork-supporters at 10 matches played at Wembley Stadium, London, or Hampden Park, Glasgow. Fieldwork-supporters observed one-way systems and signage, and hand sanitizing stations inside the stadia, but reported significant variation in the implementation of staggered timeslots, testing upon entry, and procedures for exit. Adherence to planned measures by ticket holders and implementation by stewards waned as the tournament progressed culminating in an absence of enforced measures at the final. The non-compliance with COVID-19 mitigation measures was likely to have led to a significantly increased risk of transmission. Future events should consider how COVID-19 mitigation measures could become ‘new norms’ of fan behaviour, learning from what is already known about football fandom. Tournament organizers of SMEs can use these findings to promote clearer messaging on pandemic-driven changes in fan behaviour and best practices in mitigating risk at future sporting and cultural events.

## INTRODUCTION

Sporting mega events (SMEs), defined as one-time international sporting events organized by a ‘special authority’ and generating high levels of media coverage (Byers *et al.*, 2012), potentially carry a high risk of infectious disease transmission and outbreaks because they generate large mass gatherings of people over a fixed duration (Müller, 2015; Memish *et al.*, 2019). When the SARS-CoV-2 virus (COVID-19) was declared a pandemic in March 2020 (World Health Organization, 2020a), many SMEs were postponed or cancelled (McCloskey *et al.*, 2020; Parnell *et al.*, 2020; Drury *et al.*, 2021a), as many countries had gone into lockdown, resulting in a ‘sporting standstill’ (Lee Ludvigsen, 2021a).

As restrictions eased in the spring and summer of 2021, pilot events of gradually increasing size were permitted by national governments. The UK Events Research Programme (ERP) led the way globally for the largest mass gatherings of two million people being observed at 31 sporting and cultural events (Department for Digital, Culture, Media & Sport, 2021a). The aim of the ERP was to explore ways to enable people to attend a range of events safely (Smith *et al.*, 2022). The first of three phases consisted of nine pilots in April and May 2021 and demonstrated that outdoor venues were lower risk than indoor venues, large crowds might lead to higher airborne transmission risks, and compliance with face coverings and social distancing was generally high although lower compliance was observed in higher risk areas (i.e. circulation areas) (Department for Digital, Culture, Media & Sport, 2021b). Phases 2 and 3, which consisted of 22 pilots in June and July 2021, had close to full capacities and no social distancing requirements (Department for Digital, Culture, Media & Sport, 2021a).

Postponed from the summer of 2020 to June and July 2021 (UEFA, 2020), the UEFA EURO 2020 football tournament was one of the largest SMEs to take place during the COVID-19 pandemic, culminating in the final at Wembley Stadium in England. Seven matches at Wembley formed part of the second and third phases of the ERP, coinciding with step 4 of the UK Government’s (UK Government, 2021a) ‘Roadmap’ for lifting COVID-related restrictions at large mass gathering events.^1^ Four matches also took place at Hampden Park in Scotland. However, unlike Phase 1 of the ERP, when the prevalence of the virus was low, cases were rising in the UK in the two weeks prior to the start of the tournament, with an average daily number of infections at 2392 in England and 435 in Scotland (UK Government, 2021b), despite high rates of vaccination achieved amongst adults aged 18+ in the UK (UK Government, 2021c).

Football fandom embodies a strong sense of solidarity in the way that ‘hands, arms, and bodies move in unison as part of the various supporter chants’ (Giulianotti, 2002, p. 33). Supporting as ‘normal’ carries risk in times of a pandemic due to close contact with other ticket holders, and behaviours and emotions that can exacerbate infection rates (Hopkins and Reicher, 2020). For example, physically distanced environments are at odds with the expected social norms of spectators (Templeton *et al.*, 2020). Mitigation measures to prevent virus transmission challenge the expected norms and behaviours of football fans who are conditioned to respond in a certain way, that is, to sing and shout and to celebrate a goal with excitement and passion by cheering and sometimes embracing other fans (Vallerand *et al.*, 2008). UEFA was keen to have supporters present at the EURO 2020 tournament as they are directly involved in the value creation process of football which is devalued for broadcasters when there are no fans present in the stands (Bond *et al.*, 2022). This creates tension between the established norms of attending a football match and the experience of attending a match during a pandemic. Consideration must also be given to how these measures are enforced. Whilst ‘low profile’ policing based on dialogue and facilitation (as opposed to ‘deterrence’) has previously been considered important in reducing conflict and disorder (e.g. hooliganism) and self-regulating fan behaviour at UK football matches (Stott *et al.*, 2007, 2012), little is known about COVID-19 risks and mitigation from attending an event of this scale and nature. A challenge for UEFA and host cities was whether COVID-19 mitigation measures and messaging would be accepted as ‘new norms’ of fan behaviour at matches? Thus, the aim of this paper was to examine the implementation of mitigation and messaging at UK-based EURO 2020 matches. A key objective of this research is to develop actionable insights that can support tournament organizers of future SMEs in their response to pandemic-driven changes in fan behaviour.

### Mitigation and messaging at UK-based EURO 2020 matches

Mitigation refers to the COVID-19 measures that were in place at UK-based EURO 2020 matches. A multi-layered approach to risk mitigation was necessitated (see supplementary material) due to the novel staging of the European-wide format across eleven host countries (Cowling and Aiello, 2020; Parker *et al.*, 2020; Lee Ludvigsen, 2021b), a decision made prior to the COVID-19 pandemic to celebrate the 60th anniversary of the tournament. The tournament was originally planned to take place in 13 host cities but Dublin, Ireland withdrew from hosting matches due to their inability to guarantee fans would be in attendance (a requirement from UEFA). Bilbao, Spain withdrew from the tournament to be replaced by Seville. Brussels also lost out on becoming a host city due to failure of building the Eurostadium and its four matches were subsequently awarded to London (Lovett 2017). Risk assessments of all stadia were conducted by each host city in collaboration with football associations, local government, and public health authorities (World Health Organization, 2020b). UK Government COVID-19 legislation was enforceable by law on stadia capacities, travel, isolation of confirmed cases, and quarantine of exposed persons. Stadia capacities for UK matches were calculated by the law on the social distancing requirement of 1 to 1.5 metres (Sports Ground Safety Authority Guidance, 2020). Hampden Park operated at 25% of capacity for group-stage and knockout matches and Wembley Stadium at 25% for group-stages, 50% for knockout, and 75% for the semi-final and final matches.^2^ Border restrictions required ticket holders to show proof of a negative COVID-19 test and/or quarantine for up to 10 days if travelling from countries then designated as ‘amber’ or ‘red’ list (Department for Transport and Department of Health and Social Care, 2021). COVID-19 testing was mandatory in the form of a negative lateral flow test result or proof of vaccination for ticket holders attending matches at Wembley Stadium, but not at Hampden Park (Department of Digital, Culture, Media and Sport, 2021c). Ticket holders were advised not to attend matches if they had been in close contact with someone who had tested positive for COVID-19, showed symptoms, tested positive themselves, or were required to self-isolate due to COVID-19 travel restrictions (UK Health Security Agency, 2021).

Messaging refers to how the tournament organizer (UEFA) communicated the COVID-19 mitigation measures in place at UK-based EURO 2020 matches. Ticket holders were expected to comply with UEFA’s (UEFA, 2021a) Code of Conduct, which included staggered ingress timeslots, mask-wearing, directional signage, and queuing systems. Staggered entry time slots of a 30-minute window up to 3 hours before kick-off were specified on mobile match tickets, although there was no egress protocol. Mask-wearing was mandatory by law upon entry and within stadia for matches at Hampden Park and Wembley Stadium. Changes in restrictions in England over the course of the tournament meant that mask-wearing was only required at entry and indoor areas of the stadium for knockout, semi-final, and final matches (UEFA, 2021b). Directional signage including posters, floor markings and large screens inside the ground, reinforced COVID-19 public health guidance. Hospitality, including food and drink concessions, was available at Wembley Stadium, but not at Hampden Park. One-way queuing systems were in place, although ticket holders were expected to eat and drink at their designated seats, to limit their movements at half-time and to maintain social distancing during goal celebrations.

## METHODS

This study consisted of structured observations of UEFA EURO 2020 matches played in the UK. Ethical approval was granted by the University of Stirling’s Ethics Panel (GUEP 2139). Observations were subject to a detailed risk assessment and safety protocol submitted to the Ethics Panel and approved by university staff.

### Recruitment and consent

Fieldwork-supporters were recruited through existing links with supporters’ networks, such as the Football Supporters’ Association and Supporters Direct Scotland, and social media advertising. An advert was circulated on supporters’ networks mailing lists and the project Twitter account (@EUROCOVID) inviting those who were already in possession of match tickets to be fieldwork-supporters for matches at Wembley or Hampden. Ticket holders were invited to contact the research team using the email address provided. They were then provided with an information sheet and a consent form that they could return to the research team to indicate that they wished to take part in the research.

### Sample

Eleven fieldwork-supporters (1 female and 10 males), who were already ticket holders prior to the recruitment for this study, attended 10 of the 12 EURO 2020 matches played in the UK at either Wembley Stadium, London, or Hampden Park, Glasgow. Supporters were all UK citizens (5 from England, 6 from Scotland), aged between 25 and 55 and all were experienced football fans who regularly attend either England or Scotland national team matches. In total, 7 of the 8 matches were observed at Wembley and 3 of the 4 matches at Hampden (see Table 1). There were at least two fieldwork-supporters at every match, and some attended multiple matches. Two supporters attended multiple matches due to having professional roles within UK or European organised football supporters’ networks. Consequently, 32 individual observations were recorded across the 10 matches, which, importantly, allowed for comparisons of mitigation measures at different stages of the tournament (Smith 2018). All fieldwork-supporters were given a pseudonym in the form of an ID to protect their identity.

**Table 1.** Breakdown of all matches attended and the number of fieldwork-supporters attending each match

Match	Tournament stage	Date	Permitted capacity^a^	Stadium	Fieldwork-supporter ID
England vs. Croatia (ENGCRO)	Group stages	13 June 2021	22,500	Wembley	F-S1; F-S2; F-S3; F-S4
Scotland vs. Czech Republic (SCOCZE)	Group stages	14 June 2021	12,000	Hampden	F-S5; F-S6; F-S7; F-S8; F-S9; F-S11
England vs. Scotland (ENGSCO)	Group stages	18 June 2021	22,500	Wembley	F-S12; F-S13; F-S14
Czech Republic vs. England (CZEENG)	Group stages	22 June 2021	22,500	Wembley	F-S15; F-S16; F-S17
Croatia vs. Scotland (CROSCO)	Group stages	22 June 2021	12,000	Hampden	F-S10; F-S18; F-S19; F-S20; F-S21
England vs. Germany (ENGGER)	Round of 16	29 June 2021	40,000	Wembley	F-S22; F-S23; F-S24
Sweden vs. Ukraine (SWEUKR)	Round of 16	30 June 2021	12,000	Hampden	F-S25; F-S26
Italy vs. Spain (ITAESP)	Semi-finals	6 July 2021	60,000	Wembley	F-S27; F-S28
England vs. Denmark (ENGDEN)	Semi-finals	7 July 2021	60,000	Wembley	F-S29; F-S30
England vs. Italy (ENGITA)	Final	11 July 2021	60,000	Wembley	F-S31; F-S32

^a^Maximum capacity at Wembley Stadium is normally 90,000 and 51,000 at Hampden Park.

### Data collection

All fieldwork-supporters were trained over two 2-hour online sessions on safety, data collection, and reporting procedures. The training was developed based on prior work by Fitzgerald (Fitzgerald *et al.* (2021)) and based on principles developed by Graham (Graham (2000)) and Graham (Graham *et al.*, 2009). The first training session introduced the project and the purpose of the observations. It also covered the measures in place at the EURO 2020 tournament and the safety/ethical procedures involved in carrying out the research. Session two covered the practicalities of carrying out the observation and how to fill out the observation report. Both sessions involved various interactive practical exercises developed to ensure that all participants understood the task and were comfortable with what they were being asked to do. Individualized feedback was given to each participant after they submitted their reports to clarify any areas that were not clear and/or help them to prepare for their next observation. Particular attention was given to reporting their own and others’ behaviours in relation to the mitigation measures in place at the match they were attending. Additionally, fieldwork-supporters were asked to note critical incidents which gave rise to a specific increased risk of transmission (e.g. a conflict involving ticket holders where social distancing rules were not adhered to and whether stewards intervened or not). Fieldwork-supporters were encouraged to discreetly use smartphones to type brief notes of their observations but instructed not to take pictures or videos of other ticket holders. Fieldwork-supporters were reimbursed for travel, non-alcoholic beverages and food.

A structured observation schedule (see supplementary material) was developed based on our research questions, informed by previous work (Fitzgerald *et al.*, 2021) and policy guidance from UEFA and both general COVID guidelines and measures specific to the tournament from the UK and Scottish governments. The observation schedule consisted of four sections: (i) before the match; (ii) during the match; (iii) after the match; and (iv) incident reporting. Thus, the observation schedule captured ticket holders’ behaviours in relation to mitigation measures both inside and outside the stadia (Drury *et al.*, 2021a).

Semi-structured observation reports were written up within 24 hours of each match, including detailed qualitative descriptions of relevant incidents of good practice or concern in relation to how mitigation was managed at stadia. Researchers provided individual feedback to all fieldwork-supporters by posing additional questions for clarification on observations and further elaboration or description of incident reporting.

### Analysis

All observation reports were imported into an Excel spreadsheet structured to reflect the observation schedule. We followed Braun, Clark and Weate’s (2019) six-phase thematic analysis: phases 1 and 2—familiarization and coding; phases 3, 4 and 5—theme development, refinement and naming; and phase 6—writing up. Two of the authors immersed themselves by reading through the observation reports. They then independently coded by comparing each fieldwork-supporters’ observations in relation to the overarching research question (whether COVID-19 mitigation measures and messaging would be accepted as ‘new norms’ of fan behaviour at matches?). Then the codes were thematically organized by grouping together verbatim quotes based on patterns across all observation reports. All themes were refined by iteratively comparing and reviewing how well they fit the research aim (to examine the implementation of mitigation and messaging at UK-based EURO 2020 matches). Finally, the naming of each theme was checked by all members of the research team.

## RESULTS

We present the results structured around four main themes: the measures observed to mitigate COVID-19 transmission at EURO 2020; the messaging employed to communicate measures; the perceived adherence of other ticket holders to measures and whether/how measures were enforced by stadium staff.

### Mitigation of risks associated with COVID-19

Fieldwork-supporters reported that a range of mitigation measures were in place on match days. There was a recognition that the stadia ‘haven’t been built with COVID in mind’ (F-S 12, ENGSCO) which made some measures, such as social distancing, difficult. Some fieldwork-supporters ‘felt safer entering early to avoid any late queuing’ (F-S 22, ENGGER), but for most ‘no one checked our time slot at any point’ (F-S 23, ENGGER). Fieldwork-supporters also had mixed responses to the requirement for those attending matches at Wembley to show proof of a negative lateral flow test or double vaccination against COVID alongside their match tickets. Whilst some found entry procedures ‘efficient’ (F-S 3, ENGCRO) and ‘smooth’ (F-S 22, ENGGER), two fieldwork-supporters reported that they did not present test results of vaccination at different stages of the tournament:

*I did not show my negative COVID status to anybody. The system was ineffective, and I simply walked through. (F-S 2, ENGRO)*

*[P]roof of COVID vaccination/negative test was compulsory. This unfortunately was abandoned at around 7pm, where because of the huge gatherings of crowds, to release pressure at the main entrances they let everyone through, ticket or not.* (F-S 31, ENGITA)

Inside Wembley, ticket holders queued for concession stands in a ‘cattle-pen’ (F-S 2, ENGCRO) or ‘snake formation’ (F-S 4, ENGCRO). Bright pink and yellow round floor markings of shoe silhouettes were placed on the ground to encourage social distancing (F-S 19, CROSCO). At Hampden, no concession stands were open, but seats were marked with a green ‘tick’ sticker or a red ‘cross’ sticker, and some urinals were taped off to discourage use (F-S 20, CROSCO). When seated inside the stadium bowl, adherence to mask wearing was compromised by ‘the stadium music … it was difficult for us to speak to each other without shouting at full volume’ (F-S 7, SCOCZE). As the wearing of masks is so interwoven with compliance, this mitigation measure is reported in the section on compliance with measures below. One-way systems with separate entry and exit doors were in operation at toilets, and there were green floor markings to encourage social distancing at urinals, and signs promoting good hand hygiene (F-S 18/20, CROSCO). Hand sanitizers were also located inside and outside both stadia:

*There were two [hand sanitising] stations beside every gangway entrance, one on the left and one on the right. This meant that two people could use them at once as they were moving past. There was no need for people to get in each other’s way to use the stations. Outside the stadium there were hand sanitizer stations at every entrance gate* (F-S 24, ENGGER)

However, many fieldwork-supporters did not observe any process in place for exiting the stadium (F-S 1/2/3/4, ENGCRO; F-S 5/7/8, SCOCZE; F-S 18/21, CROSCO; F-S 27, ITAESP; F-S 31, ENGITA). According to one fieldwork-supporter (F-S 3, ENGCRO), the ‘exit from the stadium was the standard procedure prior to any COVID regulations’. Consequently, maintaining social distancing became difficult on egress as fieldwork observers were ‘shoulder to shoulder with other fans as they left’ (F-S 21, CROSCO). Exiting was described as ‘[n]ot crush, but certainly usual busy squeezing into gaps that happens at the end of a normal match at Hampden’ (F-S 5, SCOCZE). Some then began to question the feasibility of entry time slots, ‘I thought it was crazy that we were all issued with staggered entry times but allowed to leave *en masse* at the end’ (F-S 19, CROSCO).

### Messaging

Messaging concerns how the mitigation measures were communicated to those attending EURO 2020 matches. Prior to attending the match, ticket holders received emails and push notification reminders via the UEFA EURO 2020 app. Purchasing a ticket indicated that ticket holders acknowledged the tournament code of conduct:

*The acknowledgment of the tournament code of conduct was signed and agreed to upon purchase of the ticket, with the most important point being that you will not attend if you have tested positive or are symptomatic. (F-S 6, SCOCZE)*

It was only announced days before the start of the tournament that proof of a negative test would be mandatory at Wembley. One fieldwork-supporter said, ‘Rules in place for Wembley were well communicated, although changed around 10 days in advance of the match, which then required a negative lateral flow test or proof of double vaccination’ (F-S 12, ENGSCO). Whilst approaching the stadium, there were posters reminding ticket holders to present proof of their negative COVID-19 test result:

*Outside the ground there were members of staff holding up signs that had the entry process on, reminding fans they had to present a negative COVID test and then their match ticket to enter the stadium perimeter. These were clear and on large poles for people to see.* (F-S 14, ENGSCO)

Posters were also placed around the concourse and on walls beside each gangway entrance containing references to mask-wearing, physical distancing and general hygiene:

*Large yellow posters ‘Protect yourself and others’ as main heading. There were 2 large images with characters and text ‘keep your distance’ [and] ‘please wear your mask at all times’. Also 3 smaller images that stressed the importance of ‘washing hands thoroughly’, ‘avoid shaking hands’ and ‘cough/sneeze into crook of your arm’*. (F-S 19, CROSCO)

Audio and visual announcements also reinforced public health guidance inside the stadium at various points during matches:

*There were three separate announcements spread across four different time periods which announced face coverings being compulsory in the seating area and walking around the stadium, to stay in your allocated seat and to ensure you follow personal hygiene guidelines*. (F-S 13, ENGSCO)

Miscommunication, however, led to some confusion about mask-wearing when UEFA sent out an email in error to ticket holders due to attend the first Scotland match at Hampden:

*The expectation was that it (mask-wearing) was required. However, over the weekend we received an email from UEFA linked to our ticket, that you could take it off whilst at your seat. Then during the match, around 70 mins, they made 2 announcements over the tannoy reminding people to keep them on. This then brought a bit of confusion as everyone had seen the ‘remove once at seat email’ which to be honest, meant nobody listened to the announcement*. (F-S 5, SCOCZE)

For matches at Wembley, the changes to allow increased capacity and reduced social distancing which occurred as part of the ERP also impacted the communication of mitigation measures for ticket holders attending matches during the knockout stages of the tournament, ‘[T]here wasn’t as many announcements as in previous games. This could have been since this was being used as a test event to get back to normal’ (F-S 22, ENGGER). Measures also changed, ‘The usual guidelines were in place that were there from previous games, however due to the attendance being increased to 60,000+, social distancing in the seating area was not required’ (F-S 27, ITAESP).

### Adherence

Adherence considers the receptiveness of ticket holders to mitigation measures. Many fieldwork-supporters felt the measures placed greater emphasis on individual responsibility (F-S 3, ENGCRO; F-S 5, SCOCZE; F-S 17, CZEENG; F-S 21, CROSCO). Although some arrived at the stadium within their allotted 30-minute time slot, most did not for various reasons:

*The timeslot given was 5:30-6:00. Several reasons for not sticking to this. Firstly, it wasn’t practical for me to get to the stadium by 5:30. Secondly, I don’t think it is reasonable to have to wait in the stadium for over 2 hours before a game. Especially on top of travel time before and after the game. In addition, the cost of doing this is prohibitive. You are very limited as the volume of food/drink you can take in, and food and drink prices are very high, so I would be more likely to waste money if I was on site for 4 or 5 hours*. (F-S 21, CROSCO)

Despite reduced stadia capacities, ‘People [were] treating it as a very normal football occasion, chatting with friends in groups, fist bumping, group photos etc…. Hard not to do given that many people will not have seen friends [for a while]’ (F-S 5, SCOCZE). Consequently, some indicated that ‘the environment encouraged breaking social distance’ (F-S 7, SCOCZE):

*I knew immediately on reading the rules that I would be breaking one of them. Tickets were allocated to our group across 2 different rows and with a gap of at least one space between all seats, and it was stated we should sit in our allocated seats. I knew that I would be sat next to my son, and whilst it wasn’t explicitly stated to ‘bunch up’ once inside the stadium it was clear this was happening all around and is a common-sense thing to do. In theory it meant there would be greater distance between bubbles*. (F-S 4, ENGCRO)

Fieldwork-supporters acknowledged that they and many others did not always adhere to measures when they were caught up in the act of supporting their team, such as when they celebrated a goal. Non-adherence to measures was increased when heightened emotions led to risky behaviours:

*It was very notable when England took the lead, all forms of social distancing went out of the window in most sections [of the stadium]... Fans were on the advertising boarding, jumping on one another and cuddling for a good couple of minutes*. (F-S 3, ENGCRO)

*The whole end section was jumping around in one massive huddle [of] hundreds/thousands of fans. I would comment that this aspect to me this looked like a ‘normal’ situation for a football game. There was no adherence or consideration to COVID guidelines at all.* (F-S 23, ENGGER)

Indeed, the occasion appeared to outweigh concerns about the risk of transmission for many ticket holders, ‘There was clearly no fear from the majority about COVID, the moment of watching England win a semi-final of a major competition for the first time in 55 years meant way more’ (F-S 31, ENGITA). Fieldwork-supporters also observed varying levels of compliance regarding mask-wearing:

*Unless people were moving to the toilets, or moving to their seats, masks weren’t* worn. (F-S 12, ENGSCO)

*It was my understanding fans still had to wear a face covering in the concourse areas when not eating or drinking, however this wasn’t adhered to … The lack of adherence was a surprise, as throughout the pandemic in my working life I was around people taking it seriously and following guidance, so it was strange to see such a lack of adherence to the guidance*. (F-S 22, ENGGER)

Some ticket holders even repurposed the ‘hand sanitiser station … as a bin effectively for beer cups’ (F-S 24, ENGGER), but the presence of hand sanitisers encouraged other fieldwork-supporters to ‘sanitise more regularly’ (F-S 22, ENGGER).

### Enforcement—response of staff

Enforcement considers which measures were enforced and by whom. There were discrepancies observed in enforcing proof of testing/vaccination at matches beyond the group stages of the tournament, despite UEFA announcing that they would do ‘spot checks … to check the vaccination/negative result status’ (F-S 27, ITAESP). Several fieldwork-supporters also noticed a lack of rigour in cross-checking ID with proof of a negative lateral flow test result, ‘I could have shown them anyone’s test. How did they know I was the person?’ (F-S 12, ENGSCO). At the semi-final and final at Wembley, ticketless fans attempted to bypass enforcement procedures altogether:

*[T]here was an increased issue of ticketless fans trying to get into the stadium. Of which a number did. They first showed their negative test, they were then in the queue for their ticket to be activated by the next steward before they entered the outer security perimeter. At this point their ‘screenshots’ of a ticket didn’t work but many decided to make a run for it into the crowded steps on the way up to the stadium. Some putting their hoods up to change their identity. At this point it looked as though these people would then not get into the stadium. However, I did see several fans ‘tailgating’ to enter the stadium. This means following another fan through the turnstile so that 2 people can enter. Several people were thrown out for this, but I imagine a number got in as well. The stewards were doing the best job they could, and it would be difficult to change that without having more [stewards] around to do so*. (F-S 29, ENGDEN)

*Many attempting to enter through the disability access doors and forcing entry as soon as they were open. All these fans did not have a ticket, nor did they have a negative COVID check. They simply bulldozed their way into the stadium*. (F-S 32, ENGITA)

The situation at the tournament final highlighted inadequate infrastructure to protect international ticket holders and stewards’ inability to enforce measures:

*[W]e witnessed fans being able to climb under the netting. There was a young steward just letting them do it, to our dismay. I and two other colleagues decided to take control, forcing England fans to go the other way. They shouldn’t have been where they were anyway, as the area they were in was the official Italian section. We watched some England fans enter the seating area and walk through the seats towards the netted area next to the bubble section. They crawled underneath the netting, upon arriving at the other side amongst the Italian fans in the bubble, the Italian fans took exception to this and started punching the England fans … control had been completely lost, and there wasn’t much we could do if the staff weren’t even able to control it … I was exhausted and furious at this point by what I had to go through and what I witnessed*. (F-S 31, ENGITA)

Indeed, this same fieldwork-supporter who tested positive for COVID-19 days after the final said, ‘[M]any fans are referring to their positive COVID tests as the ‘Wembley variant’, due to sheer number of positive tests following the game’ (F-S 31, ENGITA). Lack of enforcement has led some to question the integrity of risk mitigation, ‘I really have little faith in this system’ (F-S 21, CROSCO).

## DISCUSSION

Mitigation measures and health messaging were devised to attempt to limit virus transmission at UEFA EURO 2020 matches. Risk of transmission was dependent on how well mitigations were implemented at stadia (Murray *et al.*, 2020; Parker *et al.*, 2020; Job *et al.*, 2021; Walsh *et al.*, 2021; Bulle *et al.*, 2022). Some measures were more evident than others. For example, one-way systems, route markings and hand sanitizing stations were present inside the UK stadia utilized for the matches. However, there was clear evidence of a lack of adherence to staggered timeslots and proof of COVID testing upon entry, difficulties of mask-wearing when seated inside the stadium bowl, and no procedures to ensure social distancing on exit, despite these measures being in UEFA’s (UEFA, 2021a) code of conduct. This indicates a mismatch between the planning and implementation of mitigation measures. This is perhaps to be expected, given the unprecedented task of putting on an international tournament of 51 matches across 11 European cities during a global pandemic. It is also important to consider other factors which may have affected this implementation such as the variation in local government policies on COVID-19 restrictions and venue capacity changing during the tournament and host venues dropping out or changing location weeks before the tournament began, which had implications for recruiting and training extra staff to cover the additional matches at Wembley.

Mitigation measures were communicated by various means (Templeton *et al.*, 2020). Ticket holders were provided with information before attending EURO 2020 matches via email and push notifications from the UEFA EURO 2020 app. Messages regarding mitigation measures were also communicated via posters and announcements inside the stadia. The timing of communications about changes to mitigation was considered important, especially for promoting safe behaviours throughout the tournament (Templeton, 2021). For example, proof of a negative test/vaccination became mandatory to gain entry at Wembley days prior to the start of the tournament. Miscommunication from the tournament organizers (UEFA) led to confusion regarding the wearing of face coverings at the start of the tournament. Changes made to mitigation measures as part of the ERP (Department of Digital, Culture, Media and Sport, 2021c) also caused some confusion about messaging, with an increase in stadia capacities and fewer messages on mitigation leading many fieldwork-supporters to perceive that all mitigation measures had been abandoned.

There was less adherence to mitigation measures by ticket holders as well as a relaxing of restrictions as the tournament progressed. Unlike previous studies on ‘self-regulating/policing’ non-violent behaviours amongst football crowds (Stott *et al.*, 2007, 2012; 2020), ticket holders in this study might have been less likely to adopt the mitigation measures because the semi-finals and finals were played against the backdrop of the UK Government announcing the imminent end of COVID-related restrictions in July 2021 (Drury *et al.*, 2021b). Consequently, EURO 2020 has been described as ‘a significant risk to public health across the UK’ as it recorded the highest number of positive infections across Event Research Programme events with a total of 6376 cases identified as attending EURO 2020 matches at Wembley during the period they were likely to have acquired COVID-19 and 3036 during the period they were likely to be infectious (Smith *et al.*, 2022). It is also important to note the direct public health impact of the EURO 2020 tournament was not limited to transmission at the venue but included subsequent onward transmission, transmission to others during travel and a direct impact on the workforce related to the spread into the wider community as well as the indirect impact on the behaviour of the public through media coverage of the event (Smith *et al.*, 2022). This finding is consistent with other countries where there has been an increased incidence of COVID-19 infections as a result of attending mass sporting or other outdoor events (Alfano 2022; Suner *et al.*, 2022). These findings build on those from the ERP by offering more detailed accounts of fans’ experiences attending EURO 2020 matches in the UK, including first-hand accounts by fans who contracted COVID-19 shortly after attending matches.

Measures were differentially enforced in various stages of the tournament. Evidence of testing, which was mandatory, was often not checked, especially during later matches, and even when checked, was not cross-referenced against individuals’ ID. A key finding from the Baroness Casey Review (Casey, 2021, p. 12) of the events surrounding the EURO 2020 Final was a ‘loss of experienced stewards’ leaving ‘Wembley’s stewarding operation vulnerable’. Trust in the organizers’ ability to keep ticket holders safe, may have been further undermined by ticketless fans (Giulianotti and Klauser, 2010) gaining illegitimate entry into Wembley at semi-final and final matches, increasing the number within the stadium and risk of virus transmission.

It is important to note that, a month after the conclusion of the EURO 2020 tournament, domestic football in England and Scotland resumed with full capacity stadia. In England, ticket holders were advised that football clubs would be implementing random spot checks of their COVID-19 status at some grounds during the opening weeks (PA Media, 2021). This meant fans would either have to show proof that they had received two doses of the vaccine or proof that they had evidence of a negative lateral flow test taken within 48 hours of attending the match. Fans of Premier League clubs were also advised to comply with the Supporter Code of Conduct which included wearing masks in indoor areas, ‘avoiding close contact with people you do not know’ and following one-way signage around the stadia (Premier League, 2021a). Similar guidance was issued in Scotland. The rise of the Omicron variant in late 2021 prompted the UK Government to implement ‘Plan B’ which included the requirement for any venue of at least 10,000 capacity to check all attendees’ COVID-19 status (Premier League, 2021b). The Scottish Government also made changes to regulations in response to Omicron, at first limiting attendance at outdoor events to 500 spectators (Scottish Government, 2021) and then allowing the return to full capacity in stadia in January 2021 on the proviso that at least 50% of ticketholders (or 1000, whichever is higher) have their COVID-19 status checked, an increase from the previous target of 20%. At this time, the definition of ‘fully vaccinated’ also changed to include a booster dose (SPFL, 2022). Media reports and anecdotal evidence have since suggested low adherence from domestic football fans in relation to COVID-19 mitigation measures such as mask-wearing and social distancing, drawing criticism from other nations and concern from some managers and players (Allen, 2021; Hampson, 2021). Further research is needed to monitor the implementation of mitigation measures during the domestic football season and during future SMEs to limit the potential for virus transmission.

## STRENGTHS AND LIMITATIONS

The data gathered during this study reflect the observations, views and experiences of the fieldwork-supporters and therefore may not reflect the views and experiences of all ticket holders at UK-based EURO 2020 matches. Observations were detailed and conducted safely by fieldwork-supporters. Fieldwork supporters were briefed on what to look out for but may have missed practices or incidents of interest as they were not able to cover all areas of the stadia. Almost all fieldworkers were not from a public health background, thus lowering reporting bias.

## CONCLUSION

The redesigns of mitigation and messaging in planning and implementation are essential for limiting the risk of transmission at SMEs (Lee Ludvigsen and Parnell, 2021). Several lessons can be learned from the EURO 2020 tournament that can support tournament organizers of future SMEs in their response to pandemic-driven changes in fan behaviour and best practices in mitigating risk at future sporting and cultural events. First, ticket holders must be provided with clear, consistent and up-to-date information regarding entry (and exit) procedures and mitigation throughout the tournament and systems need to be in place to enforce any such protocols (Parker *et al.*, 2020). Second, an alternative to staggered entry may be required to avoid large queues if proof of vaccination status or proof of a negative test is to be implemented effectively at events with large attendances. Third, ticketing and entry processes need to be clear and straightforward, and information should be consistent across all communication points between the organizers and ticket holders to limit the need for multiple apps or websites and to reduce the number of checkpoints outside the stadium. Fourth, mitigation measures leave much room for improvement, with a need for clearer messaging and effective practical measures on how mitigation should be enforced within different parts of the stadia (such as mask-wearing within the stadium bowl or checking COVID-19 status upon entry) and by whom. The need for enforcement may be great at SMEs which draw ticket holders from many geographical areas. Fifth, egress procedures must be developed and implemented to ensure a safe departure from the event.

## Supplementary Material

daac176_suppl_Supplementary_Material

daac176_suppl_Supplementary_Table

## Footnotes

1 The four steps, which progressively ease restrictions over time, vary depending on the following criteria: rates of vaccination; hospitalizations and deaths; infection rates; and new Variants of Concern.

2 The increased stadia capacity in the Round of 16, semi-final and final matches at Wembley were exempted by law, as these matches became part of the ERP(Department for Business, Energy & Industrial Strategy, Department for Digital, Culture, Media & Sport and Department of Health & Social Care, 2021).
