# Supplementary material for: Attending sporting mega events during COVID-19: mitigation and messaging at UK EURO 2020 matches
Source: Health Promot Int. 2023 Jan 7;38(1):daac176. doi: 10.1093/heapro/daac176 (PMC9825819; doi:10.1093/heapro/daac176)
Supplement: daac176_suppl_Supplementary_Material [file daac176_suppl_supplementary_material.docx]

**Limiting Virus Transmission during a Sporting Mega Event (LIVE)**

**Venue Observation Report Template**

**Aim of the observations**

The EURO 2020 football tournament will be the first Sporting Mega Event (SME) since the advent of the COVID-19 pandemic. SMEs present a challenge to public health and Governments as the circulation of athletes and supporters poses issues for different risk environments. Spectators have been permitted to attend matches with appropriate mitigation measures in place (such as mask wearing, social distancing and hand sanitisation). The purpose of these observations is to monitor the COVID-19 mitigation measures in place in EURO 2020 host cities and stadia on match days.

We are focusing on 5 main areas: (A) Before the match (B) During the match (C) After the match (D) Incidents of compliance/non-compliance that are of particular interest (E) Reflections.

**Guidance about the observation notes template**

For each venue observation, please complete this ‘Report Template’ document (one report per observer per match). Please provide as much detail as you can under each section. Some of the sections have options you can chose (please circle or highlight the options that apply and/or add other information in the space provided). In other places there are open questions you should answer in your own words.

Your observer ID will be provided to you by email. **Please only use those IDs on your report.**

If you have any doubts about how to write your final observation notes, please email Jordan Maclean at: jordan.maclean1@stir.ac.uk

**Observer ID:**

**Match details**

Teams Involved: Host city:

Date: Time of kick off:

**Part A: Pre-Match Venues**

**Please add details of any venues you visited prior to attending the EURO 2020 match.**

**If you went straight to the match, skip to Part B.**

A1. What venue did you visit?


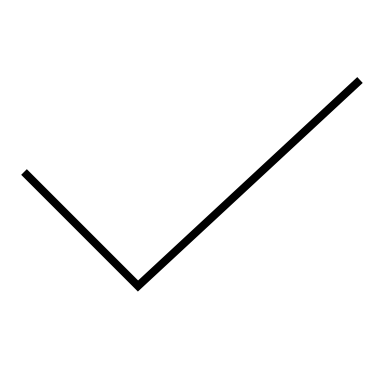
Bar pub restaurant fan zone (tick which).

Name of venue:

A2. Did you have to book in advance? Yes No

A3. Did they collect details for each household in your party? Yes No

Please give details what was collected:

A4. Were all customers were seated at tables (other than those on the way in/out or to toilets) Yes No

If No:

Some customers were seated at the bar Yes No

Some customers were standing to drink or order Yes No

Please give details:

A5. Were staff wearing?

Paper masks cloth masks high tech masks visors gloves apron

- Other: ___________________________

A6. Were the staff wearing masks properly (covering mouth & nose) and consistently (throughout the time of your observation)?

Yes No

Please give details:

A7. Were customers consistently wearing masks when not seated at tables?

Yes No, if no please describe:

A8. Did other customers maintain distancing with other tables throughout visit?

Yes No, if no please describe:

A9. Was any system in place to manage numbers in toilet areas? Yes No

Please describe:

A10. Was there music playing/television with sound? Yes No

Please describe:

**Please complete part A for each venue attended before the match.**

**Part B: At the match**

**Please add details of your experience attending the stadium**

B1: How did you travel to the stadium? (tick all travel modes that apply)

Walk Car Taxi/Uber Bus Train Underground/Tube

Coach Tram Cycle

B2. Time of entry to the stadium:

B3. Were you given a specific time period to gain entry to the stadium? Yes No

B4. Did you adhere to this time? Yes No

If No, please describe reason:

B5. Were any other guidelines/rules provided prior to you attending the match (circle as many as apply)

physical distancing face covering hygiene singing/shouting

temperature check on arrival Proof of vaccination/negative test

Tournament Code of Conduct

Other: ___________________________

B6. Were there any queues outside the stadium? Yes No

If there was a queuing system OUTSIDE the stadium, how was it managed?

spacing marked on the ground? 1m 2m

Staff/stewards telling people to keep distanced?

Posters/signage telling people not to queue?

B7. Was any of the following required in order to gain entry to the stadium?

Temperature check Proof of a negative COVID-19 test Proof of vaccination

Acknowledgment of Tournament Code of Conduct

B8. Were you shown to your seat by a member of staff/steward? Yes No

B9. Were one-way systems in operation within the stadium Yes No

If yes, how was this managed? (Circle all that applied)

Stickers/marking on the ground Posters/signage

Staff/stewards reminding people

B10. Were spectators required to wear a face covering at all times inside the stadium

Yes No

If yes, how was this managed?

Posters/signage Staff/stewards reminding people Announcements on public address system /screens

B11. Please colour the seating area of you and others around you using the box provided below. We are particularly interested in the spacing of seats between others in front and on either side of you.


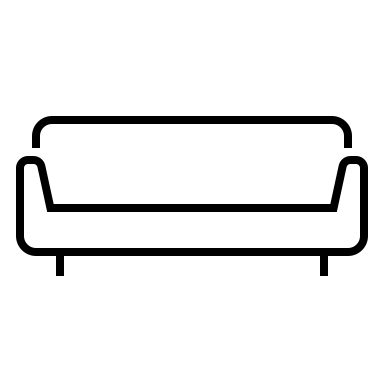

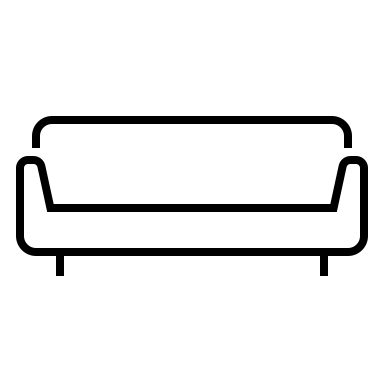

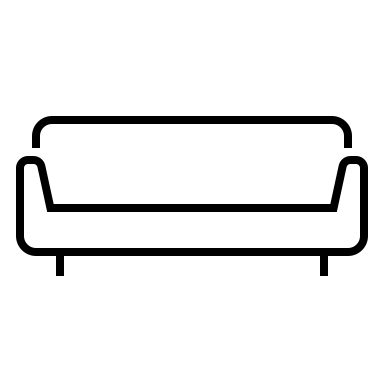

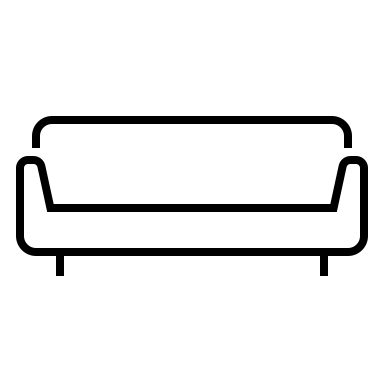

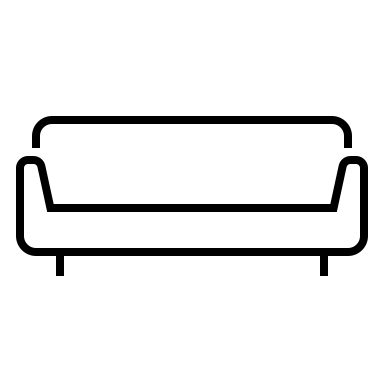

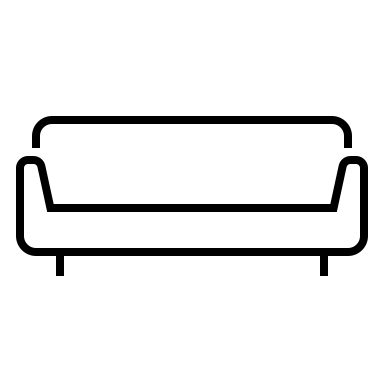

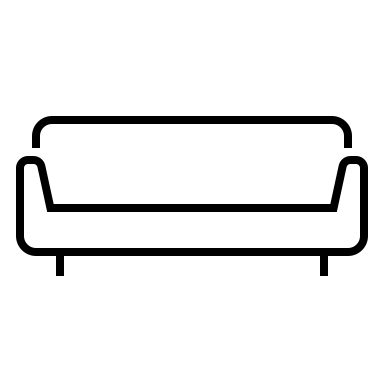

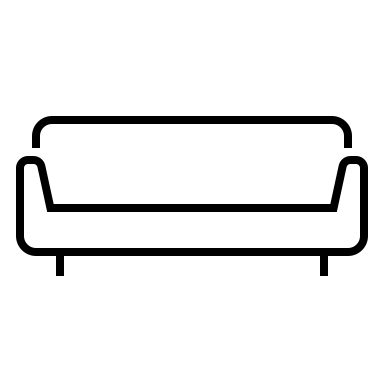

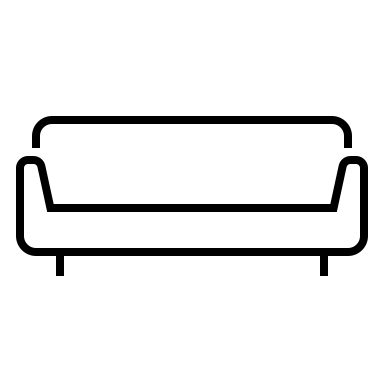

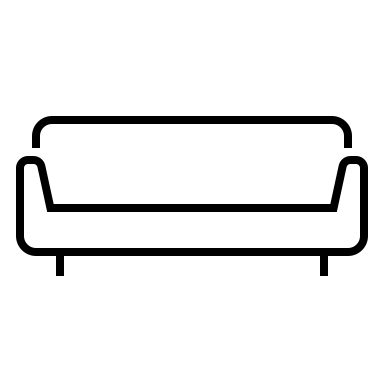

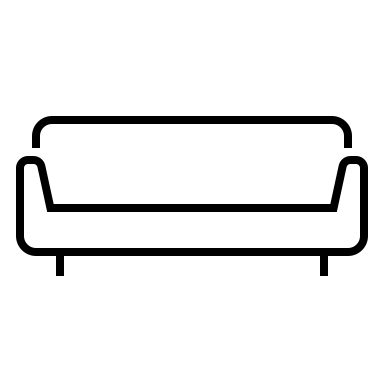

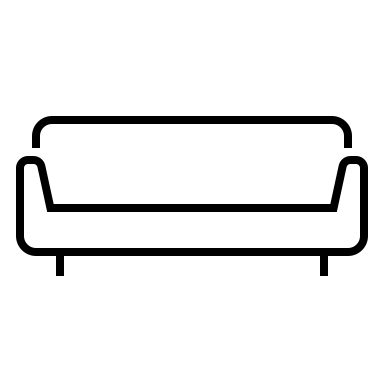


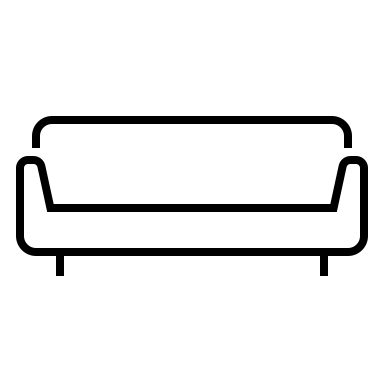

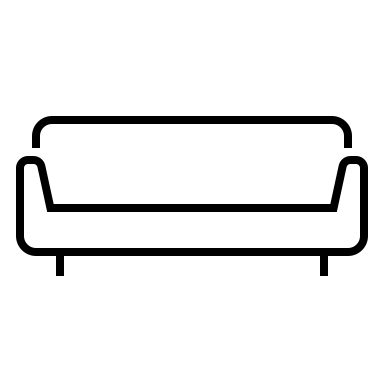

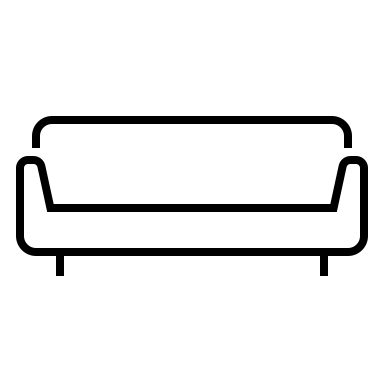

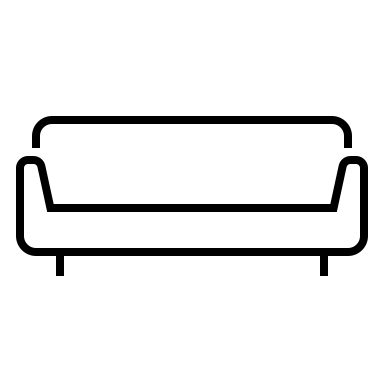

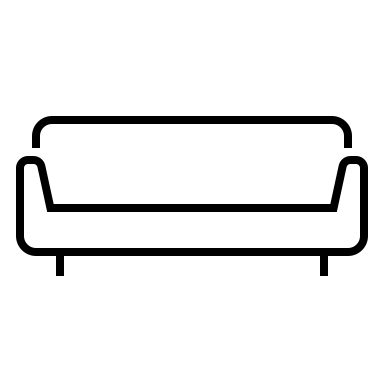

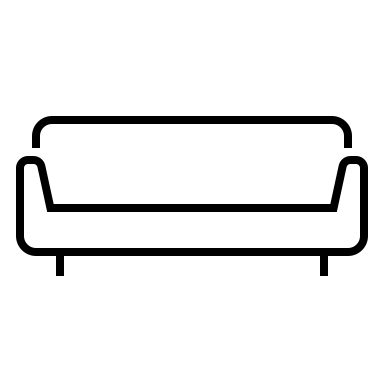

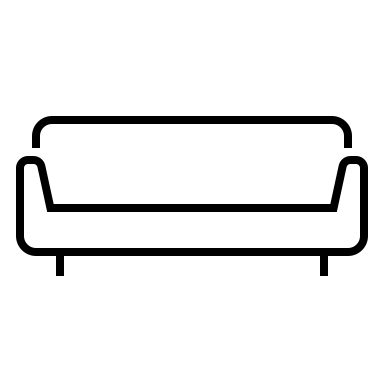

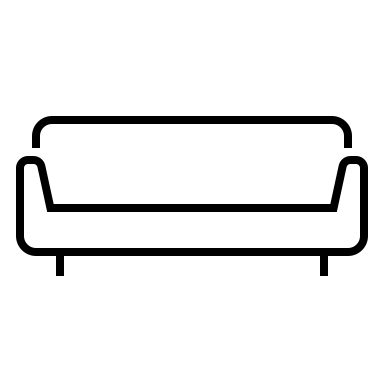

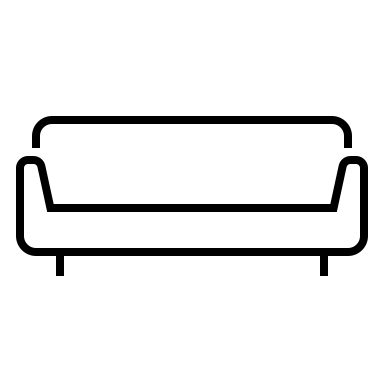

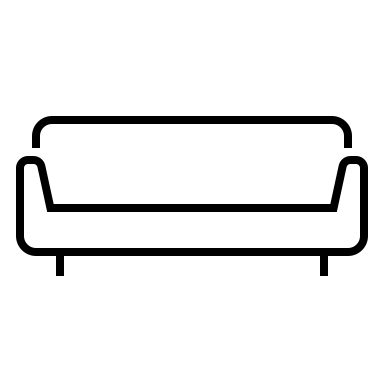

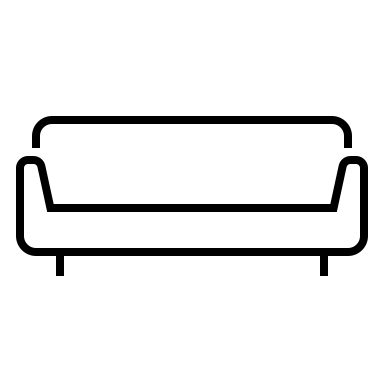

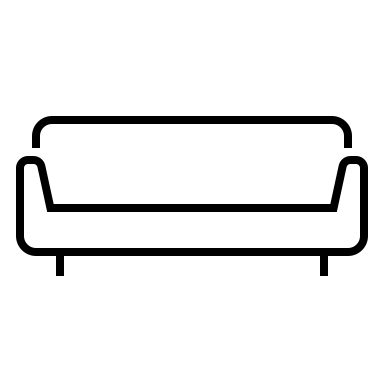


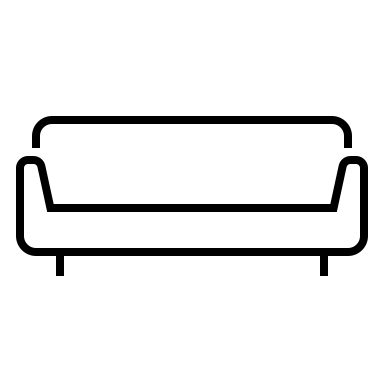

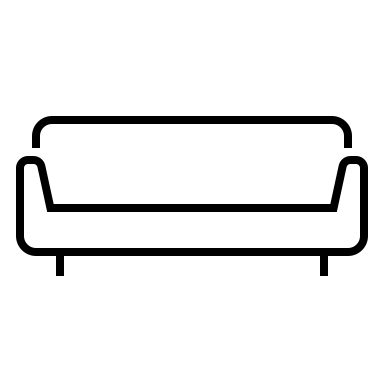

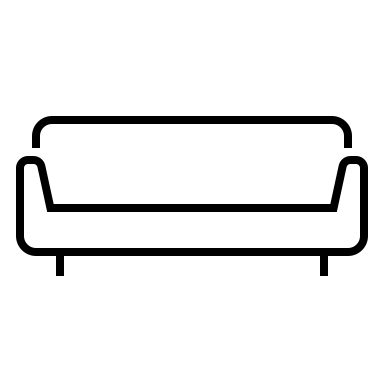

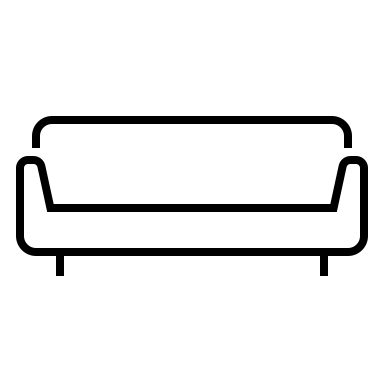

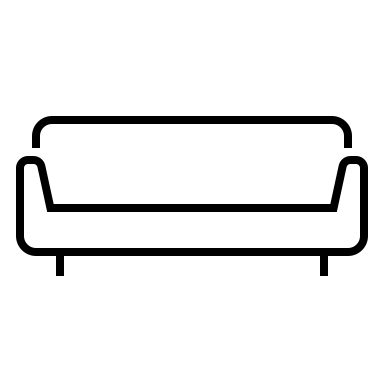

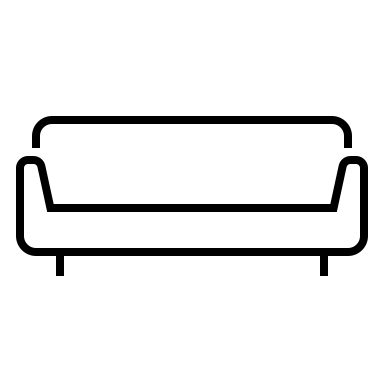

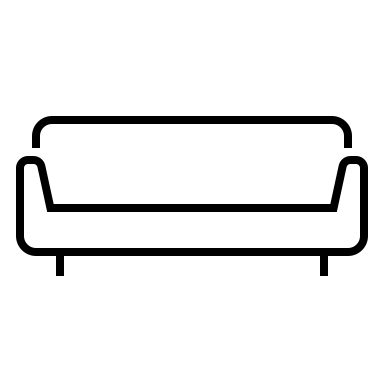

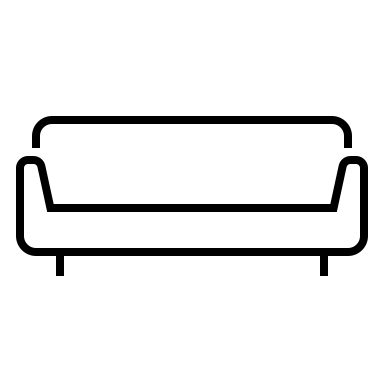

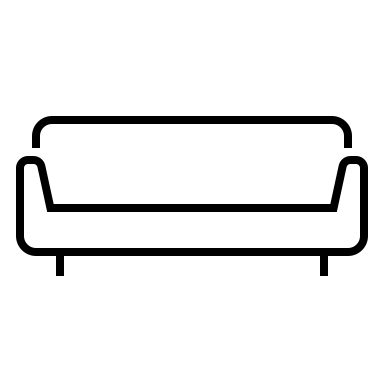

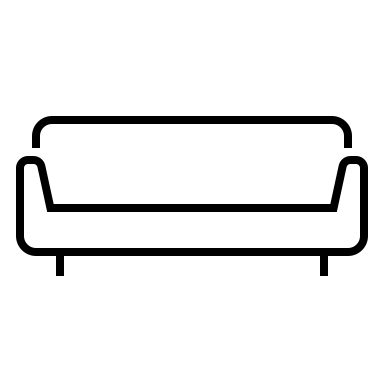

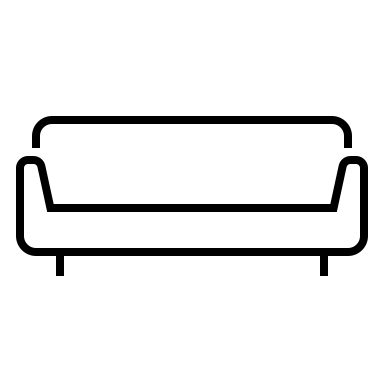

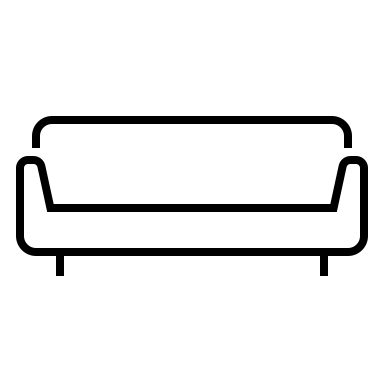


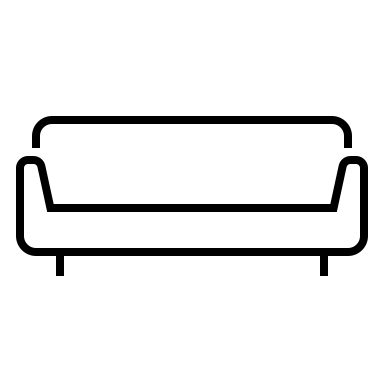

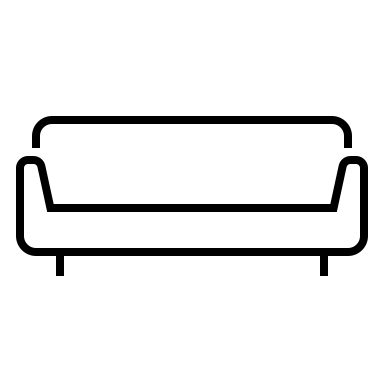

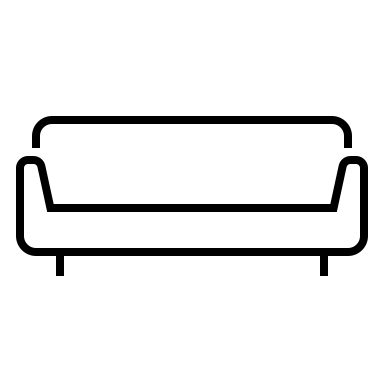

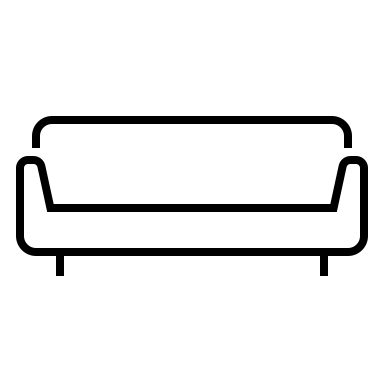

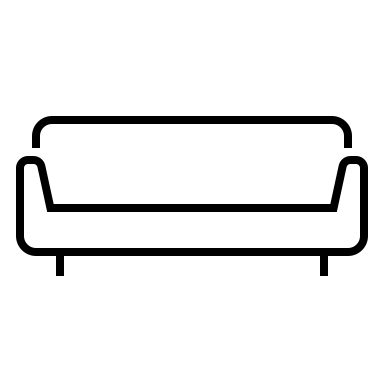

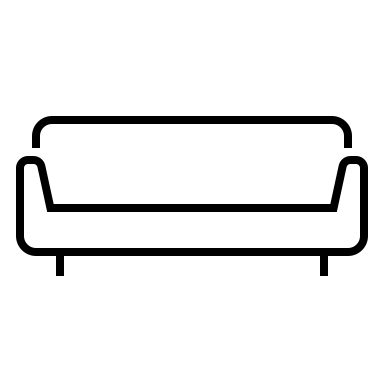

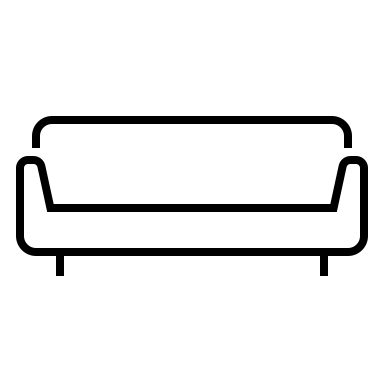

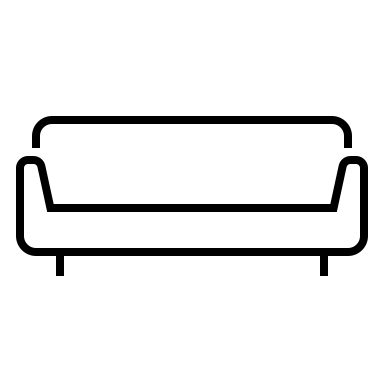

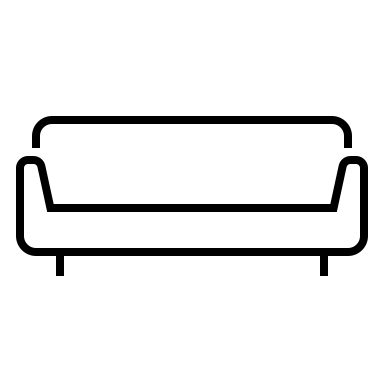

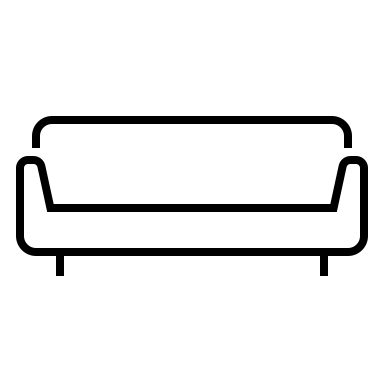

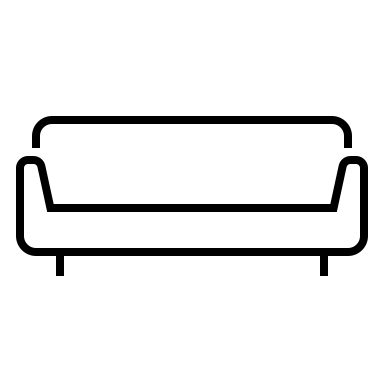

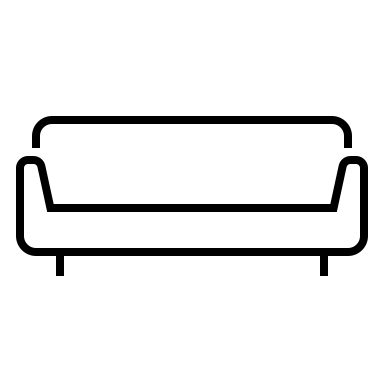


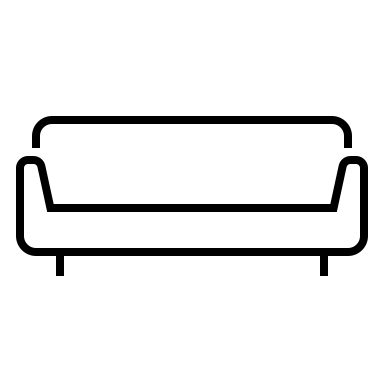

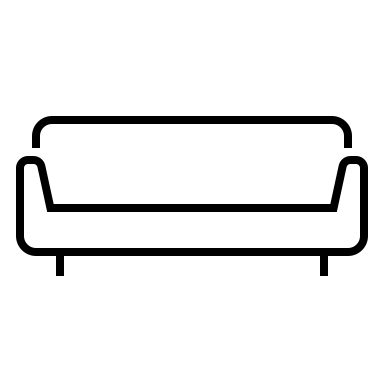

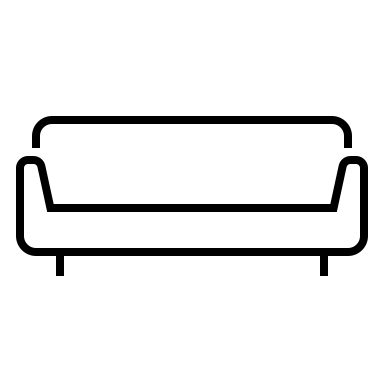

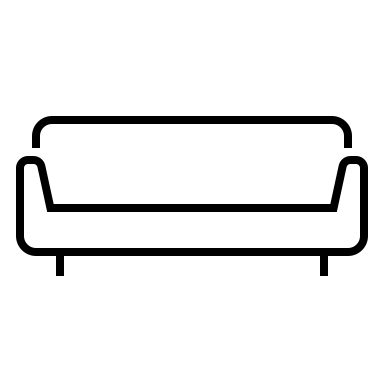

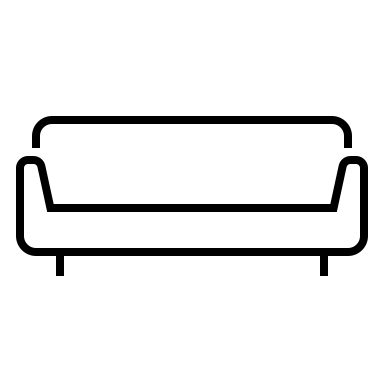

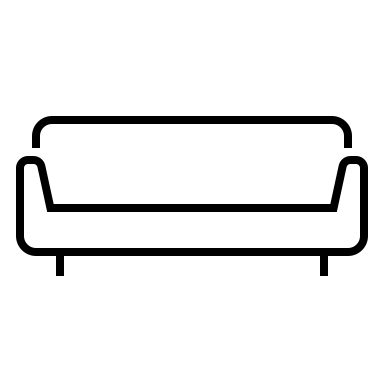

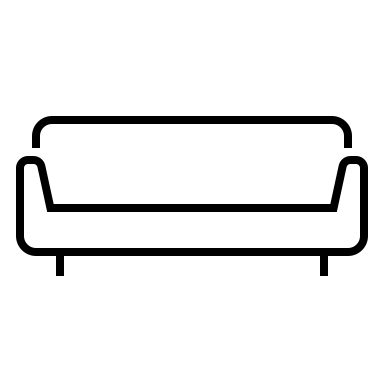

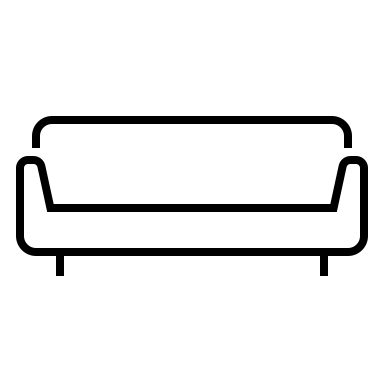

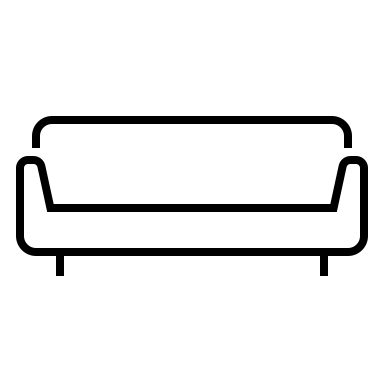

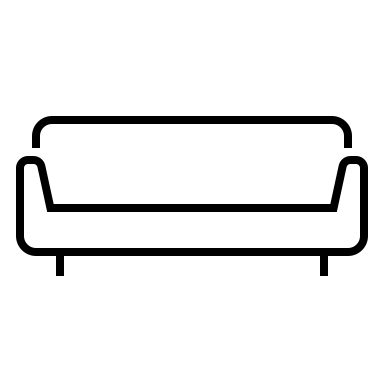

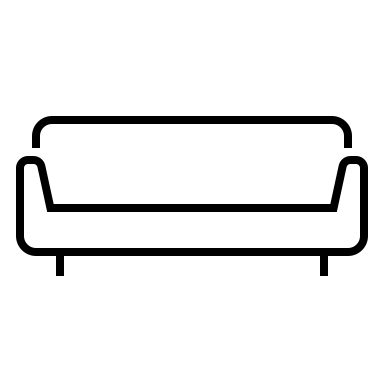

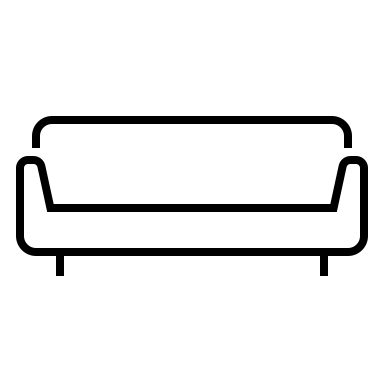


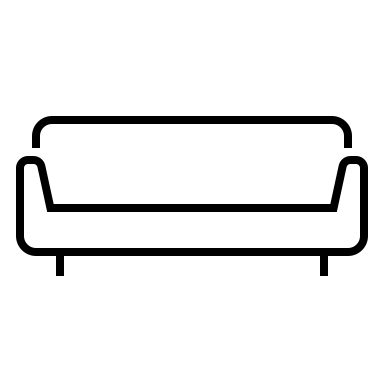

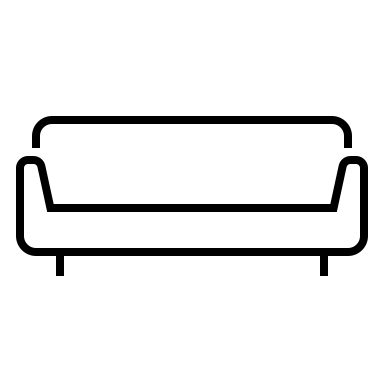

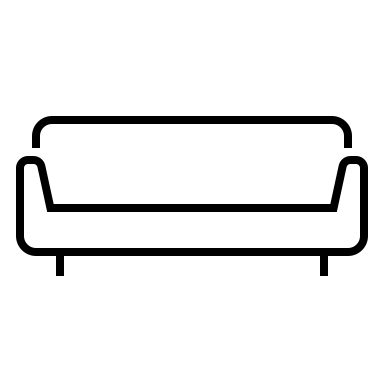

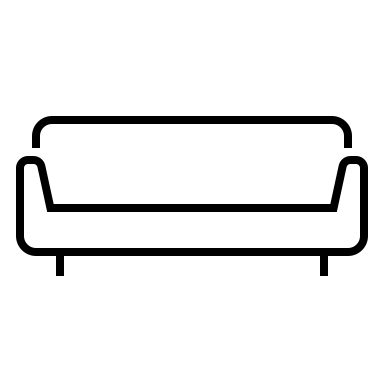

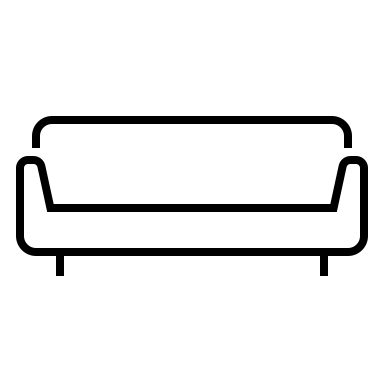

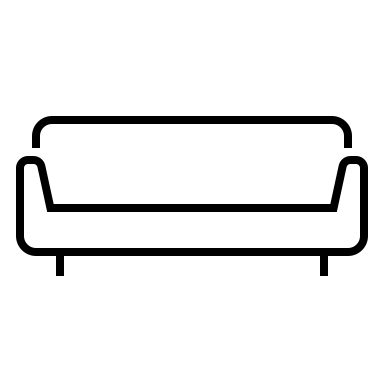

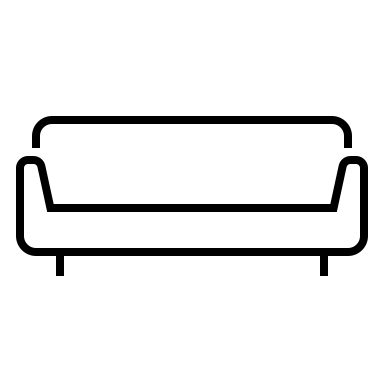

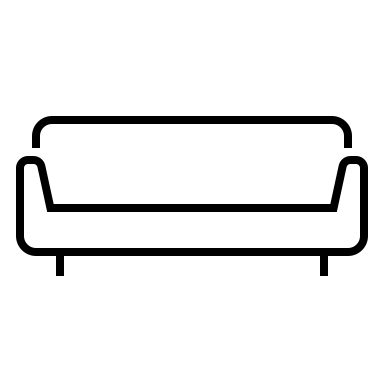

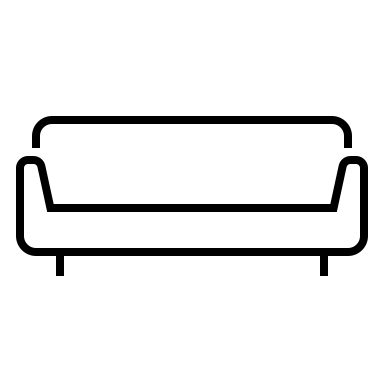

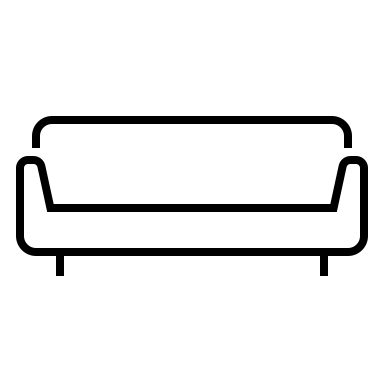

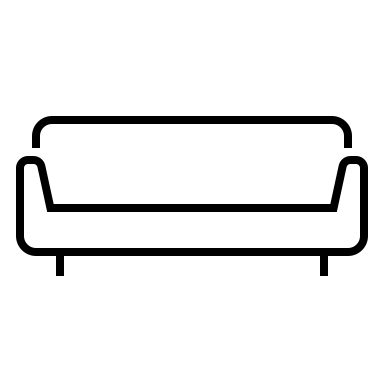

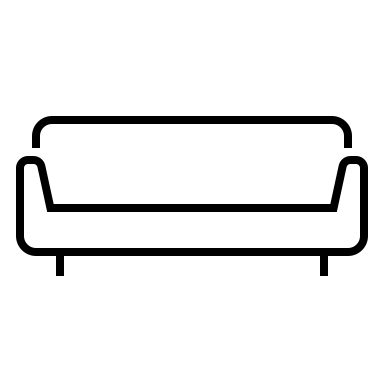


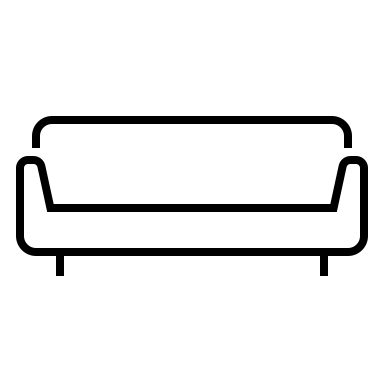

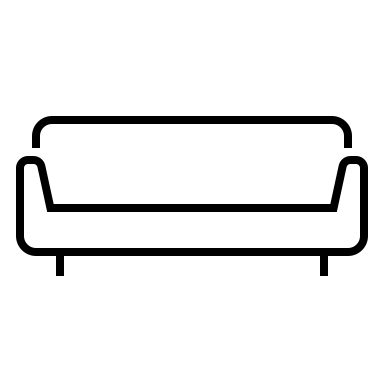

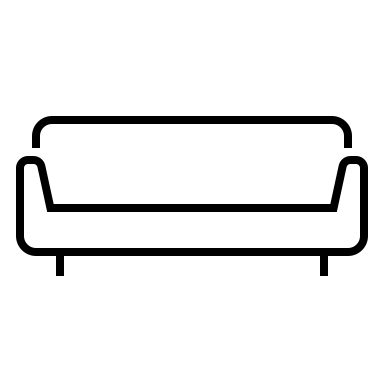

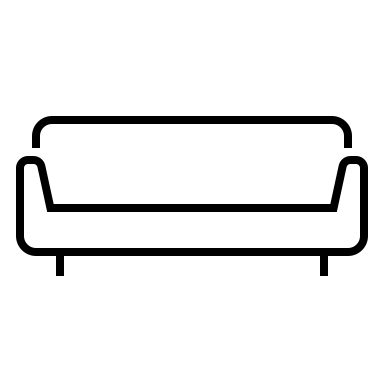

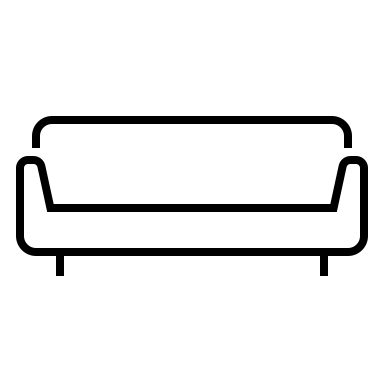

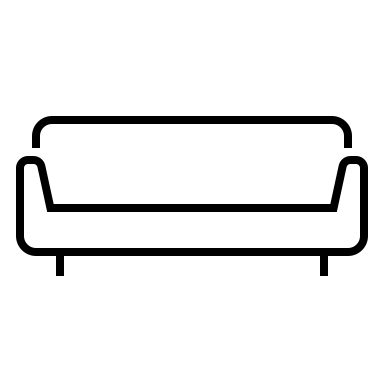

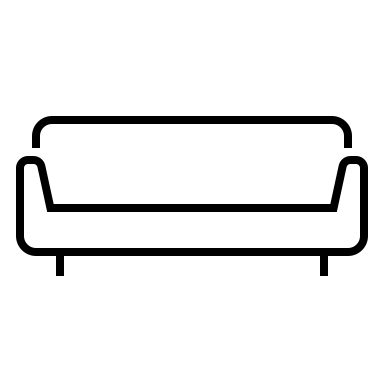

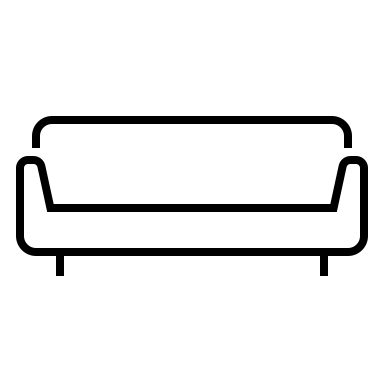

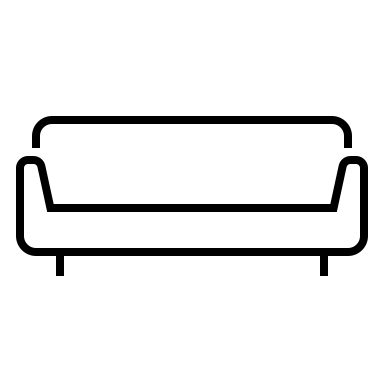

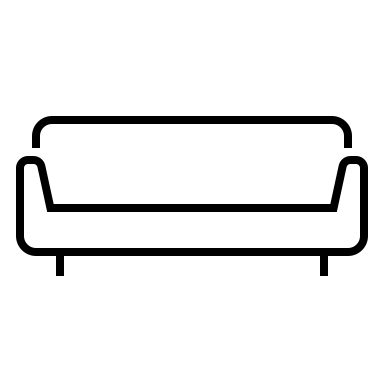

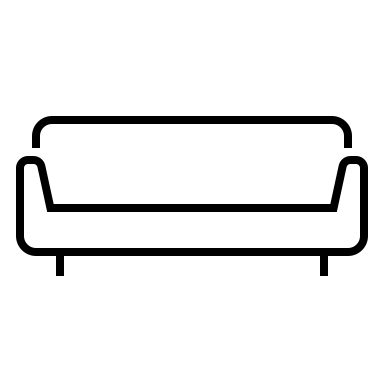

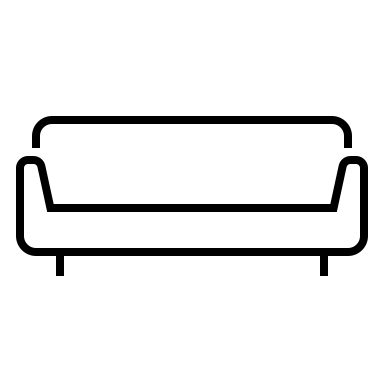


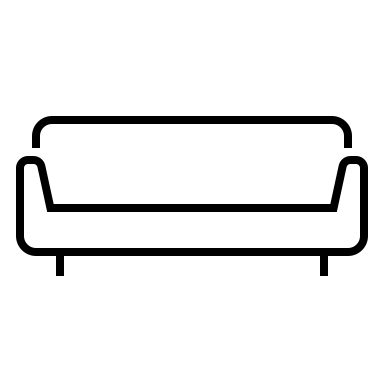

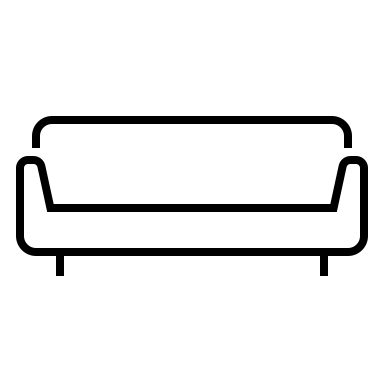

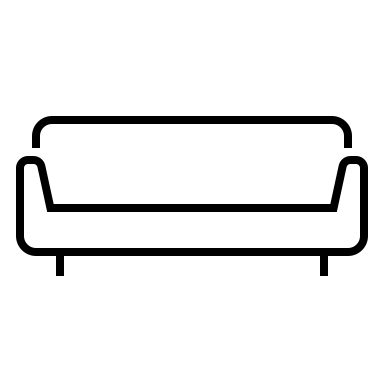

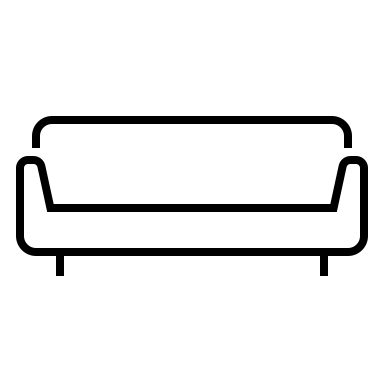

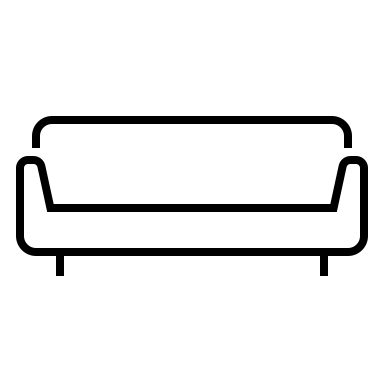

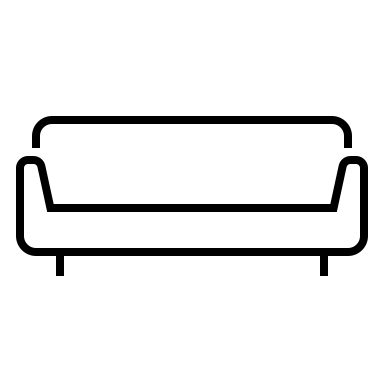

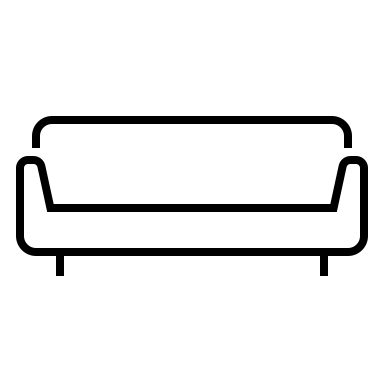

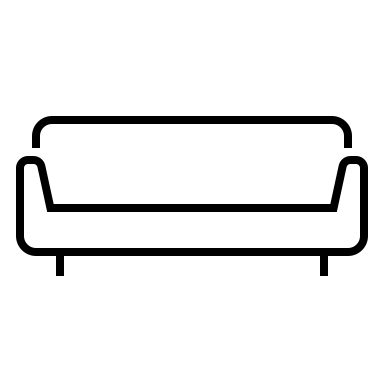

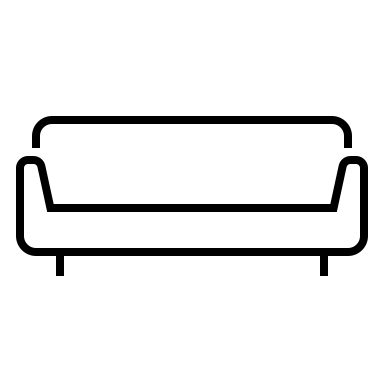

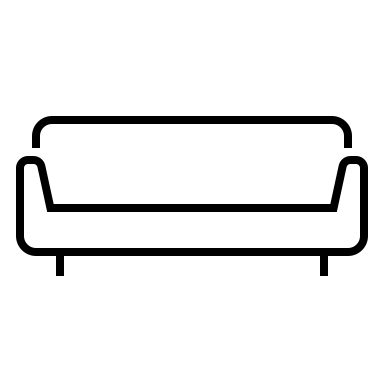

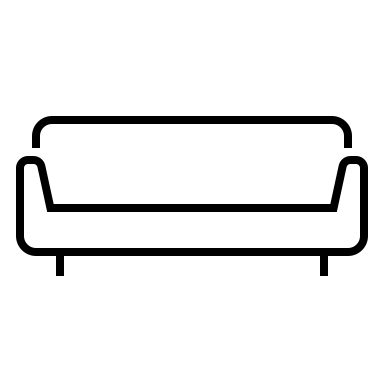

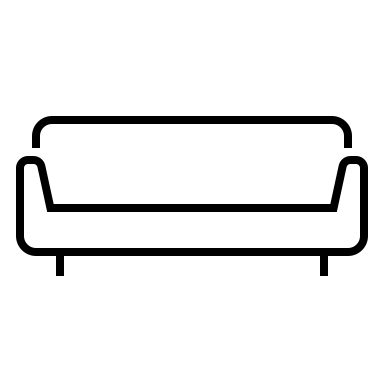


Please explain what is happening in the seating area above:

B12. Were spectators required to stay in their seats and limit their movements around the stadium? Yes No

If yes, how was this managed?

Posters/signage Staff/stewards reminding people Announcements on public address system /screens

B13. Were spectators encouraged to sanitise their hands regularly? Yes No

If yes, how was this managed?

Posters/signage Staff/stewards reminding people Announcements on public address system /screens

B14. Were spectators encouraged to keep at least 1.5 metres away from other spectators?

If yes, how was this managed?

Posters/signage Staff/stewards reminding people Announcements on tannoid/screens

B15. Was any system in place to manage numbers in toilet areas? Yes No

If yes, please describe:

B16. Were cashless payment options available to use at catering and merchandise concessions? Yes No

B17. Were there measures in place to managing queuing at catering and merchandise concessions? Yes No

Were these effective? Please describe:

B18. How was the process of exiting the stadium managed?

Staged exit in sections One-way system

If yes, how was this managed? (circle all that applied)

Stickers/marking on the ground Posters/signage

Staff/stewards reminding people

Were these effective? Please describe:

**Part C: After match**

**Please add details of any venues you visited AFTER attending the EURO 2020 match.**

**If you went straight home after the match, skip to Part D**

C1. What venue did you visit?

Bar pub restaurant fan zone (tick which).

Name of venue:

C2. Did you have to book in advance? Yes No

C3. Did they collect details for each household in your party? Yes No

Please give details what was collected:

C4. Were all customers were seated at tables (other than those on the way in/out or to toilets) Yes No

If No:

Some customers were seated at the bar Yes No

Some customers were standing to drink or order Yes No

Please give details:

C5. Were staff wearing?

Paper masks cloth masks high tech masks visors gloves apron

- Other:________________________________________

C6. Were the staff wearing masks properly (covering mouth & nose) and consistently (throughout the time of your observation)?

Yes No

Please give details:

C7. Were customers consistently wearing masks when not seated at tables?

Yes No, if no please describe:

C8. Did other customers maintain distancing with other tables throughout visit?

Yes No, if no please describe:

C9. Was any system in place to manage numbers in toilet areas? Yes No

Please describe:

C10. Was there music playing/television with sound? Yes No

Please describe:

**Please complete part C for each venue attended after the match.**

**Part D: Incident Reporting**

The primary purpose of this section is to guide you in remembering and then writing notes of as many incidents as possible that you observe that are relevant the aims of the study i.e., incidents or actions involving supporters or staff or both that illustrate one or more of the following:

- Successes/failures of the system in place in and around the stadium in terms of distancing and hygiene
- Successes/failures of the system in place in other licensed premises in terms of distancing and hygiene
- Conflict: Frustration, offensive behaviour, arguments, aggression, threatened or actual violence.

With all of these incidents we are interested in the timing and location of the incident and rich description of:

- the story of the incident – what you observe leading up to it and after
- the role of drunkenness/alcohol in the incident
- the role of covid measures/new systems in the incident and
- any involvement or potential involvement of stadium staff/stewards

**Incident 1**

- ***Time started: (****describe the incident in detail here)*
- ***Time ended:***

**Incident 2**

- ***Time started:*** ***(****describe the incident in detail here)*
- ***Time ended:***

( add more as required)

**Part E: Reflections**

In this section we ask you to reflect on your experiences of adopting a dual role at the EURO 2020 game(s): as a spectator/fan and fieldworker.

E1. Please tell us how these roles interacted with each other in both positive and negative ways before, during and after the game(s):

E2. It would also be helpful for us to know any strategies that you used to maintain the balance between being a spectator/fan and fieldworker:
