# Supplementary material for: Attending sporting mega events during COVID-19: mitigation and messaging at UK EURO 2020 matches
Source: Health Promot Int. 2023 Jan 7;38(1):daac176. doi: 10.1093/heapro/daac176 (PMC9825819; doi:10.1093/heapro/daac176)
Supplement: daac176_suppl_Supplementary_Table [file daac176_suppl_supplementary_table.docx]

**Table 2.** COVID-19 guidance comparison between Hampden Park and Wembley Stadium (Adapted from UEFA, 2021c)

|  | **Travel** | **Stadium Entry Requirements** | **COVID-19 Guidance** | **Concessions** | **Stadia Capacity** | **Exit procedure** |
| --- | --- | --- | --- | --- | --- | --- |
| Hampden Park, Scotland | If travelling outside of the Common Travel Area, adults 18 aged years or older need a negative test in three days prior to arrival; complete a Passenger Locator Form before travelling; quarantine for a minimum of five days.  Red – ten days, CV-19 test on or before day 2 and 8 of a 10-day isolation; non-red (if fully vaccinated) – no isolation if day 2 test is negative after arrival | Mobile match ticket, ID, and face mask.  No requirement to present a negative COVID-19 test or proof of vaccination. | Entry time slots: 30-minute window to arrive at stadium  Mandatory to wear a face mask unless eating or drinking  1.5 metres | No food and drink concessions | 12,000 fans, 25% of full capacity, inside the stadium | ‘Venues are currently looking at how they will approach the stadium exit procedure’ |
| Wembley Stadium, England | If travelling outside of the Common Travel Area, adults 18 aged years or older need: a negative test in three days prior to arrival; complete a Passenger Locator Form before travelling; quarantine for a minimum of five days.  Red – ten days, CV-19 test on or before day 2 and 8; Amber – same as above but with option to release after 5 days; Green – a CV-19 test on day 2 after arrival | UK residents: Mobile match ticket and ID.  All aged 11 years plus show via NHS app: 1. negative lateral flow test within 48 hours; 2. proof of full vaccination (14 days prior to match); or 3. natural immunity.  Non-UK residents: Mobile match ticket and ID.  Negative lateral flow test via text message or email within 48 hours; PCR tests from a private company or vaccination record not accepted. | Entry time slots: 30-minute window to arrive at stadium  Group stages: Mandatory to wear a face mask unless eating or drinking  Round of 16, semi-finals, and final: Face covering mouth and nose – upon entry and in all indoors areas  Social Distancing – ‘Maintain distance from others’ | Food and beverage concessions open three hours before kick-off and close ten minutes after half-time. Mitigation measures including distanced queuing and hand sanitisation | 21,500 fans for the first round of 16 match; 50% for the second round of 16 match, and 75% for the semi-finals and finals | Only specifies for entry time slots. |
